# Synthesis and Study of Some New Quinolone Derivatives Containing a 3-acetyl Coumarin for Their Antibacterial and Antifungal Activities

**Published:** 2017

**Authors:** Edris Valadbeigi, Shahram Ghodsi

**Affiliations:** *Department of Chemistry, Faculty of Sciences, Islamic Azad University, Karaj Branch, Karaj, Iran.*

**Keywords:** Antimicrobial, Chemical synthesis, Coumarin, Piperazin derivative, Quinolones

## Abstract

A series of N-[2-(8-metoxy-2H-chromen-2-one)ethyl] piperazinyl quinolones containing a carbonyl related functional groups (oxo or oxyimino) on the ethyl spacer of coumarin and piperazin rings was synthesized and studied for their antibacterial and antifungal activities . The synthesis of compounds (6a-6l) was achieved through the versatile and efficient synthetic route that involved reaction of quinolones with appropriately α- bromo ketone or α- bromo oxime derivatives (2, 2a-c). The structures of the new compounds were confirmed by IR, Mass, ^1^H-NMR and ^13^C-NMR spectra. More good activities against gram-positive and gram-negative are shown in all compounds. The antifungal data reveals that all compounds have shown weak antifungal activity as compared to Nistatin.

## Introduction

Quinoline derivatives show the main grade of heterocycles, and a number of provisions have been identified since the late 1800s. The quinoline ring system takes place in different natural products, especially in alkaloids. Derivatives of quinolones have been clinically successful and are used to care bacterial infections in either community or hospital environments. Since then, quinolones were the only synthetic agents that play a main role in the treatment of bacterial community or hospital needed illnesses. Quinolones aim bacterial type II topoisomerases, gen­erally DNA gyrase in Gram-negative bacteria and DNA topoisomerase IV in Gram-positive bacteria. Quinolines are also identified for their formation of conjugated molecules and polymers that blend improved electronic, optoelectronic, or nonlinear optical features with great mechanical properties (1-6).

Piperazine, aminopyrrolidine, and their replaced derivatives have been the most successfully applied side chains, as proved by the compounds recently on the market. The biological activity of quinoline compounds has been found to have antiasthmatic, antibacterial, anti-inflammatory, and antihypertensive properties. The quinoline shape is often used for the design of many synthetic compounds with diverse pharmacological properties. The epidemic use of these compounds and the need for clinical and pharmacological survey require fast and sensitive analytical ways for identification of its presence in biological liquid (7-12).

**Figure1:**
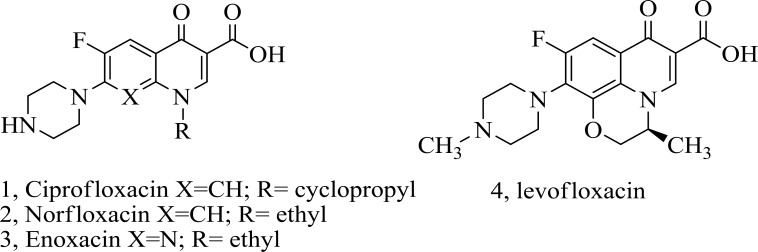
Chemical structures of some piperazinyl quinolones.

In fact, the newer fluoro­quinolones raise with the development of 7- piperazinyl quinolones, such as ciprofloxacin 1, norfloxacin 2, enoxacin 3 and levofloxacin 4 (Figure 1). The most extreme structural difference has been done on amines at the 7- position, partially due to the ease of their introduction through a nucleo­philic aromatic-substitution reaction to the related halide. We have focused our attention on modification of the C-7 basic group of the quinolone (13-18).

The coumarins inhibit ATPase activity of DNA gyrase by competing with ATP for binding to the B subunit of the enzyme. However, due to their toxicity in eukaryotes, their poor wate rsolubility, and their low activity against gram-negative bacteria, no pharmaceutically useful drug has, so far, been derived from the coumarins (19). However, renewed interest in coumarin antibiotics came from their potent gram-positive and gram-negative antibacterial activity and, which are currentlyone of the major concerns in treatment of bacterial infections (20).

In continuing our efforts to find new quinolone, we target to combine the structural characteristics of our promising antibacterial N-(2-arylethyl) piperazinyl quinolones and coumarin antibacterial drug. Therefore here, we like to state the synthesis and antibacterial activity of N-[2-(coumarin-3-yl) ethyl] piperazinyl quinolones 6 (schme 1).

*Materials and methods*

*Chemistry*

Chemical reagents and all solvents used in this research were bought from Merck AG (Darmstadt, Germany). Melting points were determined in open glass capillaries using Bibby Stuart Scientific SMP_3_ apparatus (Bibby Sterlin Ltd, U.K.) and are uncorrected. The FT-IR spectra were obtained on a Shimadzu 470 spectrophotometer (potassium bromide disks; Shimadzu, Tokyo, Japan). Mass spectra were also recorded with an Agilent Technologies 5973, Mass Selective Detector (MSD) spectrometer (Wilmigton, USA).^ 1^H-NMR spectra were recorded using a Bruker 500 spectrometer and ^13^C- NMR spectra were recorded using a Bruker 300 spectrometer (Bruker Bioscience, Billerica, MA, USA), and chemical shifts are expressed as δ (ppm) with tetramethylsilane as internal standard. Merck silica gel 60 F_254_ plates were used for analytical TLC (Merck).

## Experimental

**Scheme 1:**
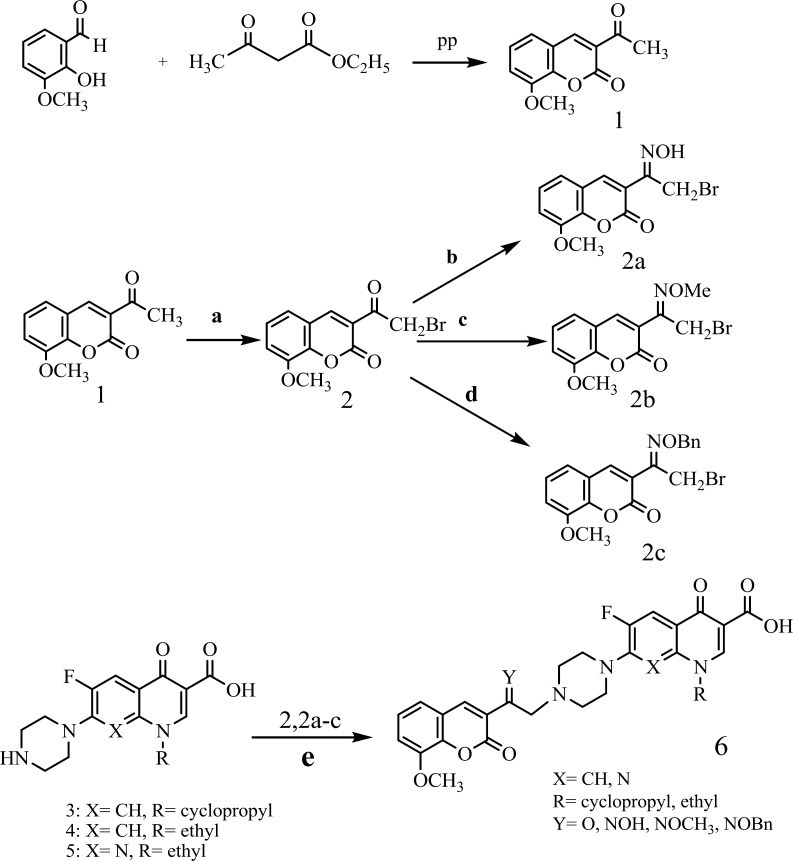
Synthesis route of compounds 6a-l.

The synthesis of N-[2-(8-metoxy-2H-chromen-2-one)ethyl] piperazinyl quinolones was obtained via the useful and effective synthetic route indicated in (Scheme 1). Then starting with 2-hydroxy-3-methoxybenzaldehyd and ethylacetoacetate in MeOH at the presence of piperidine to give 1. Compound 1 was converted to 3-(bromoacetyl) coumarin 2 by refluxing with Br_2_ in CH_2_Cl_2_ (21). Compound 2 was converted to 3- (bromoacetyl) coumarin oxime 2a by stirring with 3 equivalents of hydroxylamine hydrochloride in methanol at room temperature. Similarly, the 3-(bromoacetyl) coumarinoxime ethers 2b, 2c was obtained by reaction of compound 2 with methoxy amine hydrochloride or *O*- benzylhydroxylamine hydrochloride (22-23). Reaction of quinolones with α- bromo ketone 2 or α- bromo oxime derivatives 2a-c in DMF, at the presence of NaHCO_3_ at room temperature gives related ketones and oxime derivatives 6a-6l, respectively (Table 1) (22-23).

*Reagents and conditions*

Synthesis route of compounds, Reagents and conditions: (a) Br_2_, CH_2_Cl_2_, room temperature, and then reflux; (b) hydroxylamine hydrochloride, MeOH, room temperature; (c) methoxy amine hydrochloride, MeOH, room temperature; (d)* O*-benzyl hydroxylamine hydrochloride, MeOH, room temperature; (e) DMF, NaHCO_3_, room temperature**.**

*3-acetyl-8-methoxy-2H-chromen-2-one(1, C*_12_ *H*_10_ *O*_4_ *)*

A mixture of (400 mg, 2.6 mmol) 2-hydroxy-3-methoxybenzaldehyd and ethylacetoacetate (335 mg, 2.6 mmol) in (14 ml) MeOH at the presence of piperidine was stirred at 0-4 ºC for 4h. The precipitated yellow solid was filtered off, washed with cold methanol, and dried to give compound (1)(605 mg).Yellow solid; M.p:(168-169) ºC, yield = (78%); IR (KBr, cm^-1^):1738, 1641, 1616, 1569, 1466, 1422, 1371, 1277, 1166, 1100, 1044, 943, 890, 766; ^1^H-NMR (500 MHz, DMSO-d6): δ = 2.46 (s, 3H, CH_3_coumarin), δ = 4.07 (s, 3H, O-CH_3_coumarin), δ =7.39 (dt, 1H, H-6 coumarin, J = 7.79 and 0.81 Hz), δ =7.79 (dt, 1H, H-7 coumarin, J = 8.70 and 1.53 Hz), δ = 7.95 (dd, 1H, H-5 coumarin, J = 7.68 and 1.40 Hz), δ = 8.51 (s, 1H, H-4 coumarin) ppm; MS (70eV) *m/z*: [M^+^], 218 (100%); Anal. Calcd for C_12_H_10_O_4_ 218.2054, Found218.2051.

**Figure 2:**
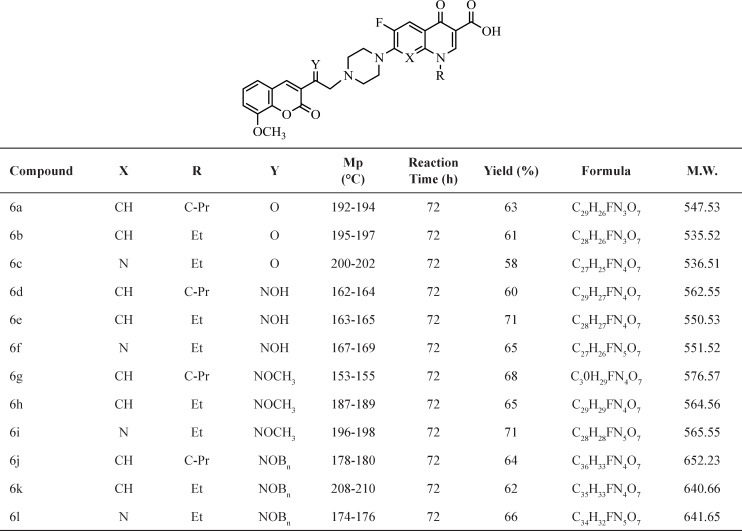
*In-vitro *antibacterial and antifungal activities of compounds 6a-l.

**Table 1:** Structures and physicochemical data of compounds 6a-l

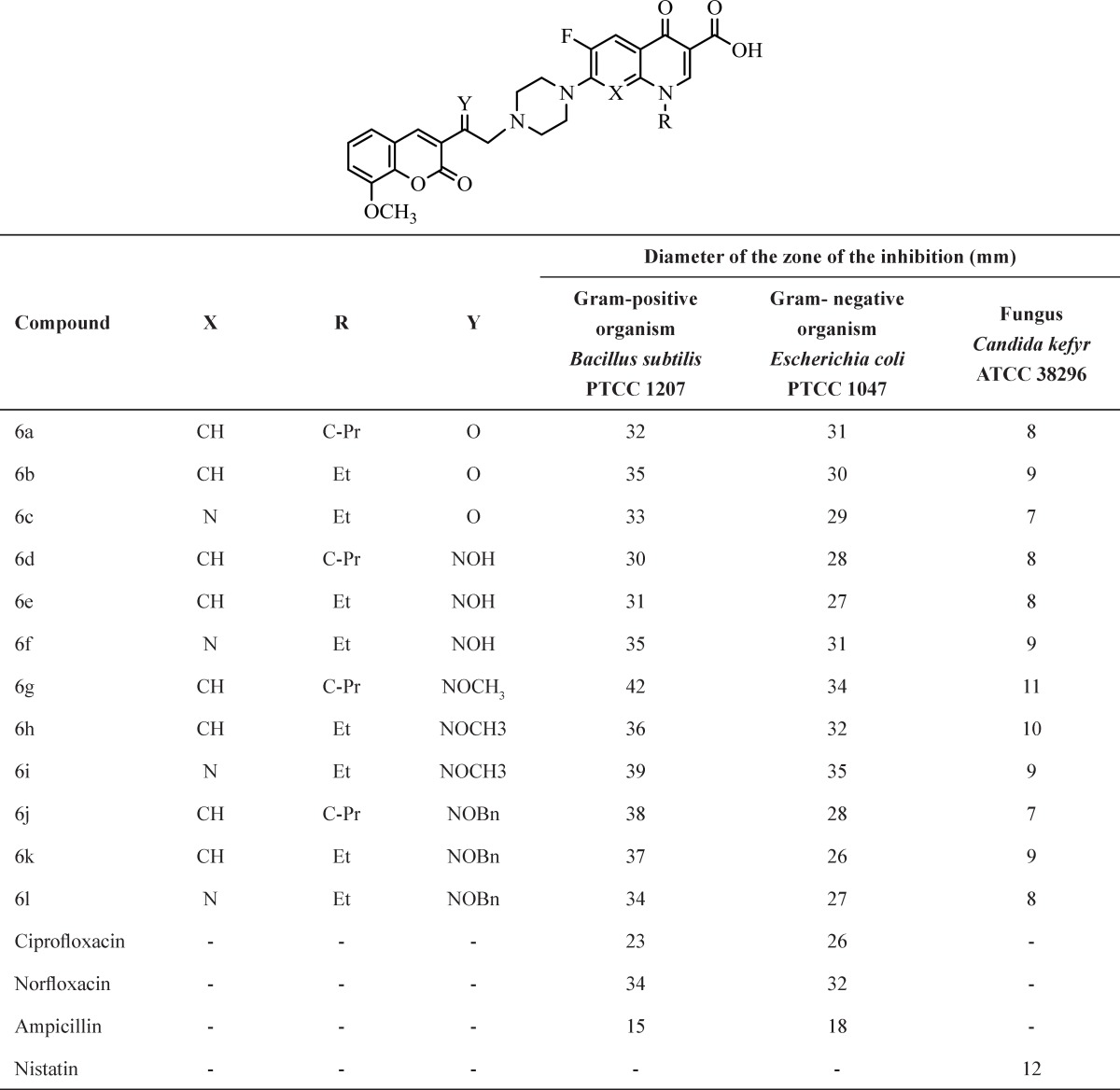

**Table 2:** *In-vitro* antibacterial and antifungal activities of compounds 6a-1.

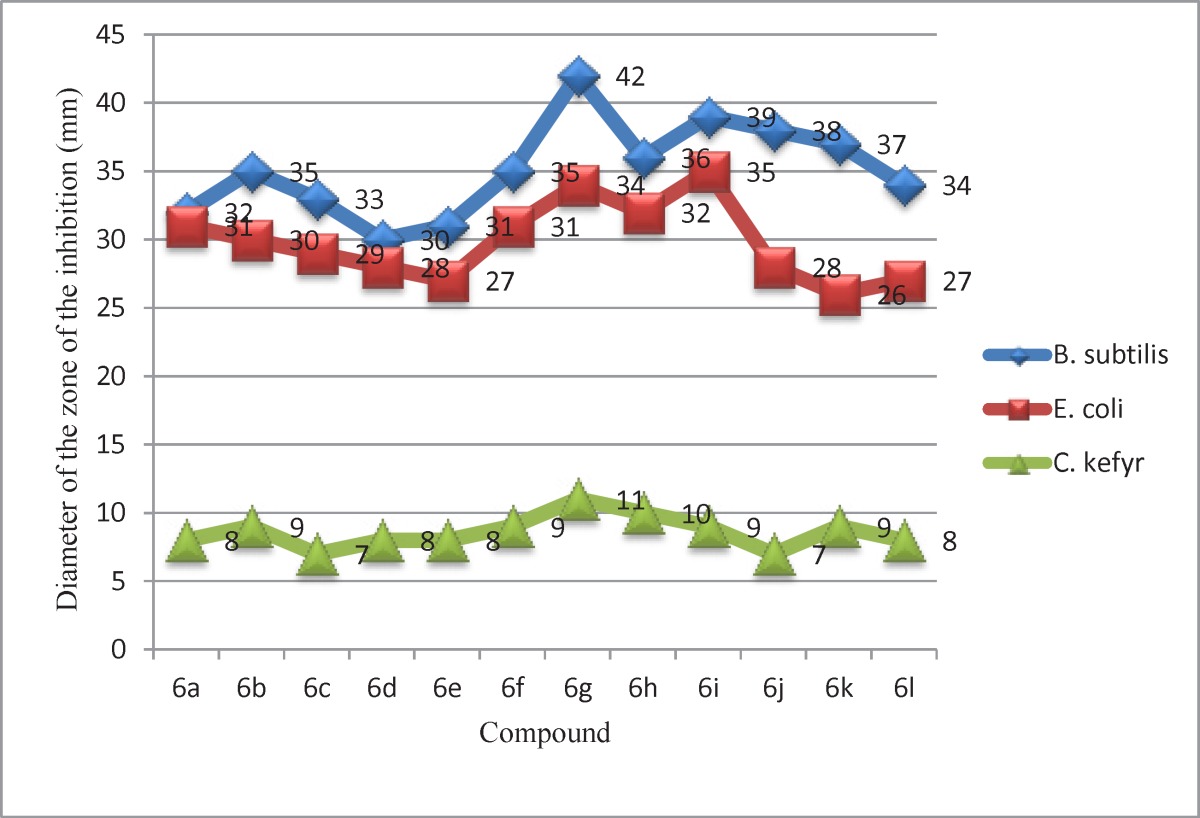

*3-(2-bromoacetyl)-8-methoxy-2H-chromen-2-one (2)*

Br_2_ (366 mg, 2.3 mmol) was added to the solution (1) (500 mg, 2.3 mmol), in CH_2_Cl_2_ a dropwise. When the reaction was completed monitored as by TLC. The precipitated yellow solid was filtered off, washed with cold methanol, and dried to give compound (2) (702 mg).Yellow crystal; M.p:(178-179) ºC, yield = (81%); IR (KBr, cm^-1^) : 1720, 1644, 1612, 1555, 1460, 1444, 1370, 1282, 1166, 1099, 1047, 1012, 890, 673; ^1^H-NMR (500 MHz, DMSO-d6): δ = 4.08 (s, 3H, O-CH_3_coumarin), δ = 4.53 (s, 2H,CH_2_-Br), δ = 7.18-7.71 (m, 2H, H-6 and H-7 coumarin), δ = 7.75 (d, 1H, H-5 coumarin, J = 8.66 Hz), δ = 8.47 (s, 1H, H-4 coumarin) ppm; MS (70eV) *m/z*: [M^+^], 295 (100%), [M^+^ +2], 297(30%); Anal. Calcd for C_12_H_9_^79^BrO_4_ 297.1015, Found297.1006.

*3-(2-bromo-1-(hydroxyimino) ethyl)-8-methoxy-2H-chromen-2-one (2a)*

A solution of (2) (297 mg, 1.0 mmol) and hydroxylamine hydrochloride (209 mg, 3.0 mmol) in MeOH (10 mL) was stirred at 22-25 ºC for 24 h. Then, water (25 mL) was added and the precipitate was filtered and washed with water to give compound (2a) (389 mg). White powder; M.p:(165-167) ºC, yield = (77%); IR(KBr, cm^-1^): 1730, 1723, 1677, 1615, 1444, 1361, 1258, 1171, 1025, 955, 835, 760;^1^H-NMR (500 MHz, DMSOd6): δ = 4.06 (s, 3H, O-CH_3_ coumarin), δ= 4.41 (s, 2H, CH_2_-Br), δ =7.51 (dt, 1H, H-6 coumarin, J = 7.91 and 0.88 Hz), δ =7.72 (dt, 1H, H-7 coumarin, J = 8.68 and 1.56 Hz), δ =7.93 (dd, 1H, H-5 coumarin, J = 7.71 and 1.40 Hz), δ =8.33 (s, 1H, H-4 coumarin), δ =12.39 (s, 1H, oxime) ppm; MS (70eV) *m/z*: [M^+^], 310 (100%), [M^+^ +2], 312(20%); Anal.Calcd for C_12_H_10_^79^BrNO_4_ 312.1161, Found312.1164.

*3-(2-bromo-1-(methoxyimino) ethyl)-8-methoxy-2H-chromen-2-one (2b)*

25% solutionof *O*-methyl hydroxyl ammonium chloride was added to a stirred solution of (2) (297 mg, 1.0 mmol) in MeOH (16 ml) at 22-25 ºC in diluted HCl (1002 mg, 3.0 mmol). After 24 h stirring at 22-25 ºC, the precipitated white solid was filtered off, washed with cold methanol, and dried to give compound (2b)(987 mg). White powder; M.p: (141-143) ºC, yield = (76%); IR (KBr, cm^-1^): 1726, 1636, 1601, 1576, 1461, 1429, 1368, 1251, 1123, 1097, 1053, 1014, 891, 761; ^1^H-NMR (500 MHz, DMSO-d6): δ = 4.06 (s, 3H, O-CH_3 _coumarin), δ = 4.25 (s, 2H,CH_2_-Br), δ = 4.15 (s, 3H, O-CH_3_ oxime), δ =7.39 (t, 1H, H-6 coumarin, J = 7.42 Hz), δ =7.62-7.89 (m, 2H, H-5 and H-7 coumarin), δ =8.21(s, 1H, H-4 coumarin) ppm; MS (70eV) *m/z*: [M^+^], 324 (100%), [M^+^ +2], 326(25%); Anal.Calcd for C_13_H_12_^79^BrNO_4_ 326.1427, Found326.1421.

*3-(1-(benzyloxyimino)-2-bromoethyl)-8-methoxy-2H-chromen-2-one (2c)*

A solution of (**2**) (297mg, 1.0 mmol) and *O*-benzyl hydroxyl amine hydrochloride (0.479 g, 3.0 mmol) in MeOH(16 mL) was stirred at 22-25 ^º^C for 24 h. The Outcome was cooled (0-4 ^º^C) and the precipitated white solid was filtered off, washed with MeOH, and dried to give compound (2c) (527) mg. White powder; M.p:(105-107) ^º^C, yield = (68%); IR (KBr, cm ^-1^) :1722, 1617, 1615, 1445, 1357, 1247, 1158, 1105, 1048, 877, 771, 728; ^1^H-NMR (500 MHz, DMSO-d6): δ = 3.76 (s, 3H, O-CH_3_ coumarin), δ = 4.44 (s, 2H, CH_2_-Br), δ = 5.41 (s, 2H, O-CH_2_-Ph), δ = 7.28-7.55 (m, 7H, H-6, H-7, coumarin and phenyl), δ = 7.78 (d, 1H, H-5 coumarin. J = 7.72), δ = 8.00 (s, 1H, H-4 coumarin) ppm; MS (70eV) *m/z*: [M^+^], 401 (100%), [M^+^ +2], 403(28%); Anal. Calcd for C_19_H_16_^79^BrNO_4_ 402.2386, Found402.2379.

*General procedure for the synthesis of compounds 6a-l*

A mixture of 3-(bromoacetyl-8-methoxy) coumarin 2 or 3-(bromoacetyl-8-methoxy) coumarin oxime derivatives 2a-c (0.55 mmol), quinolone 3-5 (0.5 mmol), and NaHCO_3_ (0.5 mmol) in DMF (5 mL), was stirred at room temperature for three days. After consumption of quinolone, when the reaction was completed monitored as by TLC, water (30 mL) was added and the precipitate was filtered, washed with water, and recrystallized from EtOH-CHCL_3_ (9:1) to give the target compounds 6a-l.

*1-cyclopropyl-6-fluoro-7-(4-(2-(8-methoxy-2-oxo-2H-chromen-3-yl)-2-oxoethyl) piperazin-1-yl)-4-oxo-1, 4-dihydroquinoline-3-carboxylic acid(6a)*

White powder; M.p: (192-194) ^º^C, yield = (63%);IR (KBr, cm^-1^):3449, 1731, 1690, 1628, 1610, 1560, 1475, 126; ^1^H-NMR ( DMSO-d6 , 500 MHz ): δ = 1.14 -1.28 (m, 2H, cyclopropyl),δ = 1.29-1.30 (m, 2H, cyclopropyl),δ = 2.48 -2.55(m, 4H, piperazine), δ = 3.21-3.43 (m, 4H, piperazine), δ = 3.87 (s, 3H, O-CH_3_coumarin), δ = 4.19 (s, 2H, C-CH_2_-N), δ = 4.25 (m, 1H, cyclopropyl), δ = 7.12 (s, 1H, H-8 quinolone, J = 7.42 Hz), δ = 7.21 (t, 1H, H-7 coumarin, J = 7.76 Hz), δ = 7.49 (d, 1H, H-6 coumarin, J = 8.35 Hz), δ = 7.54 (m, 1H, H-5 coumarin, J = 7.79 and1.46 Hz), δ = 7.91 (d, 1H, H-5 coumarin , J = 1.36 Hz), δ = 8.04 (s, 1H, H-5 quinolone), δ = 8.53 (s, 1H, H-4 coumarin ), δ = 8.67 (s, 1H, H-2 quinolone), 15.35(s, 1H, COOH) ppm; ^13^C-NMR (DMSO-d6, 300 MHz): δ = 7.8 (2C), 38.7, 39.2, 39.8, 53.1(2C), 56.1, 58.3 (2C), 66.5, 104.8, 111.2, 113.1, 116.7, 119.1, 119.9, 131.6, 133.7, 138.1, 139.1, 148.7, 149.1, 150.1, 153.4, 164.2, 166.3, 172.2, 198.1 ppm.

*1-ethyl-6-fluoro-7-(4-(2-(8-methoxy-2-oxo-2H-chromen-3-yl)-2-oxoethyl) piperazin-1-yl)-4-oxo-1, 4-dihydroquinoline-3-carboxylic acid (6b)*

White powder; M.p:(195-197) ^º^C, yield = (61%); IR (KBr, cm^-1^): 3438, 1725, 1633, 1615, 1563, 1475, 1460, 1379, 1256, 1195, 968, 758; ^1^H-NMR (500 MHz, DMSO-d6) : δ =1.45 (t, 3H,CH_3_, J = 7.21 Hz), δ = 2.68-2.80 (m, 4H, piperazine), δ = 3.29 -3.42 (m,4H, piperazine), δ = 3.91 (s, 3H, O-CH_3_coumarin), δ = 3.96 (s, 2H, COCH2), δ = 4.62 (q, 2H, CH_2_-CH_3_, J = 7.09 Hz), δ = 7.22 (d, 1H, H-8 quinolone, J = 7.32 Hz), δ = 7.38 (t, 1H, H-6 coumarin, J = 7.52 Hz), δ = 7.81 (dt, 1H, H-7 coumarin, J = 7.09 and 1.59 Hz), δ = 7.92 (d, 1H, H-5 quinolone, J = 13.26 Hz), δ = 8.00 (dd, 1H, H-5 coumarin, J = 7.86 and 1.41 Hz), δ = 8.51 (s, 1H, H-4 coumarin), δ = 8.85 (s, 1H, H-2 quinolone), δ =15.45 (s, 1H, COOH) ppm;^ 13^C-NMR (DMSO-d6, 300 MHz): δ = 15.5, 52.7, 53.1 (2C), 56.8, 57.3 (2C), 61.4, 106.5, 112.2, 114.1, 116.2, 118.3, 119.5, 126.5, 130.6, 133.7, 142.1, 145.8, 149.1, 149.8, 151.4, 152.5, 165.2, 167.3, 172.4, 179.8, 198.5 ppm.

*1-ethyl-6-fluoro-7-(4-(2-(8-methoxy-2-oxo-2H-chromen-3-yl)-2-oxoethyl) piperazin-1-yl)-4-oxo-1, 4-dihydro-1, 8-naphthyridine-3-carboxylic acid (6c)*

White powder; M.p:(200-202) ^º^C, yield = (58%); IR (KBr, cm^-1^): 3428, 1738, 1691, 1634, 1605, 1566, 1471, 1451, 1257, 962, 814, 754; ^1^H-NMR (500 MHz, DMSO-d6): δ = 1.40 (t, 3H, CH_3_, J = 7.12 Hz), δ = 2.70 -2.78 (m, 4H, piperazine), δ = 3.84-3.89 (m, 4H, piperazine), δ = 3.88 (s, 3H, O-CH_3_coumarin), δ = 3.96 (s, 2H, COCH_2_), δ = 4.51 (q, 2H, CH_2_-CH_3_, J = 7.10 Hz), δ = 7.38 (t, 1H, H-6 coumarin, J = 7.35 Hz), δ = 7.80 (dt, 1H, H-7 coumarin, J = 7.81 and 1.39 Hz), δ = 8.02 (dd, 1H, H-5 coumarin, J = 7.76 and 1.21 Hz), δ = 8.16 (d, 1H, H-5 quinolone, J = 13.49 Hz), δ = 8.58 (s, 1H, H-4 coumarin), δ = 8.91 (s, 1H, H-2 quinolone), δ = 15.30 (s, 1H, COOH) ppm; ^13^C-NMR (DMSO-d6, 300 MHz): δ = 15.3, 50.8 (2C), 53.1, 56.6, 57.4 (2C), 65.8, 111.8, 113.6, 115.9, 119.4, 120.4,126.4, 132.1, 133.9, 137.3, 139.8, 149.1, 149.8, 152.1, 153.4, 164.8, 166.6, 174.4, 178.8, 199.3 ppm.

*1-cyclopropyl-6-fluoro-7-(4-(2-(hydroxyimino)-2-(8-methoxy-2-oxo-2H-chromen-3-yl) ethyl) piperazin-1-yl)-4-oxo-1, 4-dihydroquinoline-3-carboxylic acid (6d)*

White powder; M.p:(162-164) ^º^C, yield = (60%); IR (KBr, cm^-1^): 3431, 1718, 1632, 1462, 1390, 1344, 1258, 755;^ 1^H-NMR (DMSO-d6, 500 MHz ): δ = 1.05-1.21 (m, 2H, cyclopropyl), δ = 1.26-1.35 (m, 2H, cyclopropyl), δ = 2.04 -2.49 (m, 4H, piperazine), δ = 3.25-3.33(m, 4H, piperazine), δ = 3.77(s, 3H, O-CH_3 _coumarin), δ = 4.12 (m, 1H, cyclopropyl), δ = 4.21 (s, 2H,C-CH_2_-N), δ = 7.08 (s, 1H, H-8 quinolone, J = 8.32 Hz), δ = 7.29 (t, 1H, H-7 coumarin, J = 7.62 Hz) , δ = 7.46 (d, 1H, H-6 coumarin, J =7.34 Hz), δ = 7.70 (m, 1H, H-5 coumarin, J = 7.35 and 1.31 Hz), δ = 7.99 (s, 1H, H-4 coumarin, J = 7.69 Hz), δ = 8.10 (d, 1H, H-5 quinolone, J =13.47 Hz), δ = 8.60 (s, 1H, H-2 quinolone), 15.38(s, 1H, COOH) ppm; ^13^C-NMR (DMSO-d6, 300 MHz): δ = 7.8 (2C), 38.1, 39.4, 43.3, 53.8(2C), 55.9, 57.4 (2C), 61.7, 102.4, 106.7, 111.8, 114.2, 118.4, 119.7, 124.6, 124.9, 130.1, 134.2, 135.9, 145.6, 147.9, 153.3, 158.8, 165.2, 166.9, 187.1 ppm. 

*1-ethyl-6-fluoro-7-(4-(2-(hydroxyimino)-2-(8-methoxy-2-oxo-2H-chromen-3-yl) ethyl) piperazin-1-yl)-4-oxo-1, 4-dihydroquinoline-3-carboxylic acid (6e)*

White powder; M.p:(163-165) ^º^C, yield = (71%); IR (KBr, cm^-1^): 3436, 1725, 1661, 1633, 1478, 1469, 1382, 1251, 810, 765; ^1^H-NMR (500 MHz, DMSO-d6): δ = 1.35 (t, 3H, CH_3_, J = 7.17 Hz), δ = 2.56- 2.66 (m, 4H, piperazine), δ = 3.23-3.33 (m, 4H, piperazine), δ = 3.51 (s, 2H, CNOH-CH_2_), δ = 3.91 (s, 3H, O-CH_3_coumarin), δ = 4.61 (q, 2H, CH_2_-CH_3_, J = 7.25 Hz), δ = 7.11 (d, 1H, H-8 quinolone, J = 7.36Hz), δ = 7.38 (t, 1H, H-6 coumarin, J = 7.81 Hz), δ = 7.70 (d, 1H, H-7 coumarin, J = 7.81 Hz), δ = 7.83 (d, 1H, H-5 coumarin, J = 7.73 Hz), δ = 7.96 (d, 1H, H-5 quinolone, J = 13.32 Hz), δ = 8.06 (s, 1H, H-4 coumarin), δ = 8.93 (s, 1H, H-2 quinolone), δ = 11.28 (s, 1H, oxime), δ =15.31 (s, 1H, COOH) ppm;^ 13^C-NMR (DMSO-d6, 300 MHz): δ = 15.1, 52.8, 53.4 (2C), 56.9, 57.8 (2C), 62.3, 108.5, 112.8, 114.9, 117.5, 119.4, 126.8, 128.4, 131.2, 140.9, 144.6, 146.7, 148.9, 155.3, 159.8, 162.4, 164.2, 167.9, 172.5, 186.6 ppm. 

*1-ethyl-6-fluoro-7-(4-(2-(hydroxyimino)-2-(8-methoxy-2-oxo-2H-chromen-3-yl) ethyl) piperazin-1-yl)-4-oxo-1, 4-dihydro-1, 8-naphthyridine-3-carboxylic acid (6f)*

White powder; M.p:(167-169) ^º^C, yield = (65%); IR (KBr, cm^-1^): 3429, 1722, 1638, 1451, 1380, 1271, 812, 768; ^1^H-NMR (500 MHz, DMSO-d6): δ = 1.41 (t, 3H, CH_3_, J = 7.25 Hz), δ = 2.70-2.76 (m, 4H, piperazine), δ = 3.68-3.76 (m, 4H, piperazine), δ = 3.81 (s, 2H, CNOH-CH_2_), δ = 3.92 (s, 3H, O-CH_3_coumarin), δ = 4.55 (q, 2H, CH_2_-CH_3_, J = 7.08 Hz), δ =7.46 (t, 1H, H-6 coumarin, J = 7.72 Hz), δ = 7.64 (dt, 1H, H-7 coumarin, J = 7.66 and 1.38 Hz), δ = 7.78 (d, 1H, H-5 coumarin, J = 7.80 Hz), δ = 8.08 (d, 1H, H-5 quinolone, J = 13.52 Hz), δ = 8.26 (s, 1H, H-4 coumarin), δ = 8.89 (s, 1H, H-2 quinolone), δ = 11.38 (s, 1H, oxime), δ =15.38 (s, 1H, COOH) ppm;^13^C-NMR (DMSO-d6, 300 MHz): δ = 15.6, 50.5 (2C), 53.8, 56.1, 57.2 (2C), 64.9, 113.5, 114.8, 116.1, 119.3, 122.5, 126.2, 133.3, 134.5, 139.8, 144.3, 149.1, 149.8, 152.1, 158.3, 164.8, 167.9, 174.5, 177.4, 187.5. ppm.

*1-cyclopropyl-6-fluoro-7-(4-(2-(8-methoxy-2-oxo-2H-chromen-3-yl)-2-(methoxyimino) ethyl) piperazin-1-yl)-4-oxo-1, 4-dihydroquinoline-3-carboxylic acid (6g)*

White powder; M.p:(153-155) ^º^C, yield = (68%);IR (KBr, cm^-1^): 3449, 1733, 1630, 1599, 1488, 1460, 1340, 1260, 1050; ^1^H-NMR (DMSO-d6, 500 MHz ) : δ = 1.12 -1.23(m, 2H, cyclopropyl), δ = 1.23-1.30 (m, 2H, cyclopropyl), δ = 2.08 - 2.36 (m, 4H,piperazine), δ = 3.30-3.41 (m, 4H, piperazine), δ = 3.91(s, 3H,O-CH_3_coumarin), δ = 3.97 (s, 3H, O-CH_3 _oxime), δ = 4.16 (m, 1H, cyclopropyl), δ = 4.26 (s, 2H, C-CH_2_-N), δ = 7.08 (s, 1H, H-8 quinolone), δ = 7.30 (s, 1H, H-6 coumarin, J = 7.52 Hz), δ = 7.47 (d, 1H, H-7 coumarin, J = 8.62 Hz), δ =7.56 -7.84 (m, 1H, H-5coumarin J = 7.33 and 1.26 Hz), δ = 7.99 (s, 1H, H-4 coumarin), δ = 8.12 (d, 1H, H-5 quinolone, J = 13.12 Hz), δ = 8.63(s, 1H, H-2 quinolone), 15.29(s, 1H, COOH) ppm;^ 13^C-NMR (DMSO-d6, 300 MHz): δ =7.8 (2C), 37.3, 39.1, 44.2, 53.6(2C), 56.3, 57.6 (2C), 62.1, 65.2, 102.5, 106.7, 112.8, 114.2, 118.4, 119.7, 124.6, 124.8, 130.2, 134.1, 136.9, 146.1, 147.6, 152.4, 158.8, 165.5, 166.7, 185.2 ppm.

*1-ethyl-6-fluoro-7-(4-(2-(8-methoxy-2-oxo-2H-chromen-3-yl)-2-(methoxyimino) ethyl) piperazin-1-yl)-4-oxo-1, 4-dihydroquinoline-3-carboxylic acid (6h)*

White powder; M.p:(187-189) ^º^C, yield = (65%); IR (KBr, cm^-1^): 3459, 1728, 1630, 1524, 1481, 1449, 1388, 1262, 1055, 878, 771; ^1^H-NMR (500 MHz, DMSO-d6): δ = 1.65 (t, 3H, CH_3_, J = 7.18 Hz), δ = 2.66-2.76 (m, 4H, piperazine), δ = 3.11 -3.18 (m, 4H, piperazine), δ = 3.89 (s, 3H, O-CH_3_coumarin), δ = 3.99 (s, 2H, C-CH_2_-N), δ = 4.04 (s, 3H, OCH_3_oxime), δ = 4.27(q, 2H, CH_2_-CH_3_, J = 7.21 Hz), δ = 6.82 (d, 1H, H-8 quinolone, J = 6.79 Hz), δ = 7.38 (t, 1H, H-6 coumarin, J = 6.88 Hz), δ =7.49-7.71 (m, 2H, H-5 and H-7 coumarin), δ = 7.97 (s, 1H, H-4 coumarin), δ = 8.11 (d, 1H, H-5 quinolone, J = 13.11 Hz), δ = 8.69 (s, 1H, H-2 quinolone), δ = 15.16 (s, 1H, COOH) ppm;^ 13^C-NMR (DMSO-d6, 300 MHz): δ = 15.6, 54.8, 55.4 (2C), 57.9, 58.8 (2C), 62.3, 66.8, 109.8, 112.1, 115.1, 118.5, 120.8, 127.8, 129.2, 131.2, 143.3, 145.6, 147.6, 149.3, 155.6, 159.8, 163.8, 165.9, 167.3,170.3, 173.4, 184.8 ppm. 

*1-ethyl-6-fluoro-7-(4-(2-(8-methoxy-2-oxo-2H-chromen-3-yl)-2-(methoxyimino) ethyl) piperazin-1-yl)-4-oxo-1, 4-dihydro-1, 8-naphthyridine-3-carboxylic acid (6i)*

White powder; M.p:(196-198) ^º^C, yield = (71%); IR (KBr, cm^-1^): 3451, 1728, 1638, 1476, 1278, 1133, 1040, 1018, 892, 811, 756; ^1^H-NMR (500 MHz, DMSO-d6): δ = 1.44 (t, 3H, CH_3_, J = 7.22 Hz), δ = 2.58-2.71 (m, 4H, piperazine), δ = 3.71 -3.78 (m, 4H, piperazine), δ = 3.87 (s, 3H, O-CH_3_coumarin), δ = 3.96 (s, 2H, C-CH_2_-N), δ = 4.05(s, 3H, O-CH_3_oxime), δ = 4.46 (q, 2H, CH_2_- CH_3_, J = 7.25 Hz), δ = 7.43 (dt, 1H, H-6 coumarin, J = 7.65 and 0.81 Hz), δ = 7.62 -7.71 (m, 2H, H-5 and H-7 coumarin), δ = 7.99 (s, 1H, H-4 coumarin), δ = 8.18 (d, 1H, H-5 quinolone, J = 13.44 Hz), δ = 8.1 (s, 1H, H-2 quinolone), δ = 15.09 (s, 1H, COOH) ppm; ^13^C-NMR (DMSO-d6, 300 MHz): δ = 15.7, 50.2 (2C), 52.9, 55.8, 56.9 (2C), 60.4, 63.5, 112.5, 115.7, 117.2, 119.6, 123.4, 128.6, 133.6, 135.1, 138.7, 145.5, 148.8, 149.1, 154.6, 157.9, 165.4, 169.8, 174.8, 176.6, 183.6 ppm.

*7-(4-(2-(benzyloxyimino)-2-(8-methoxy-2-oxo-2H-chromen-3-yl) ethy) piperazin-1-yl)-1-cyclopropyl-6-fluoro-4-oxo-1,4-dihydroquinoline-3-carboxylic acid (6j)*

White powder; M.p:(178-180) ^º^C, yield = (64%); IR (KBr cm^-1^): 3440, 1733, 1631, 1502, 1471, 1345, 1263, 1036, 1008, 876, 762, 719;^ 1^H-NMR (DMSO-d6, 500 MHz): δ = 1.17-1.22 (m,2H, cyclopropyl), δ = 1.25 -1.31 (m, 2H, cyclopropyl), δ = 2.36 -2.49 (m, 4H, piperazine), δ = 3.22 -3.37 (m, 4H, piperazine), δ = 3.79 (s, 3H, O-CH_3_coumarin), δ = 4.11 (m, 1H, cyclopropyl), δ = 4.12 (s, 2H, C-CH_2_-N), δ = 5.11 (s, 2H, O-CH_2_-Ph), δ = 7.15 (s, H-8 quinolone ), δ = 7.26-7.67 (m, 7H, H-6 coumarin, H-7 coumarin and phenyl), δ = 7.54-7.61 (m, 1H, H-5 coumarin J = 7.29 and 1.30 Hz), δ = 7.89 (s, 1H, H-4 coumarin), δ = 8.12 (d, 1H, H-5 quinolone, J = 13.10 Hz), δ = 8.76 (s, 1H, H-2 quinolone), 15.29(s, 1H, COOH) ppm;^ 13^C-NMR (DMSO-d6, 300 MHz): δ = 7.9 (2C), 37.5, 39.6, 45.2, 54.2(2C), 56.4, 58.3 (2C),65.5,80.1, 104.7, 111.2, 113.1, 116.7, 117.7, 119.2, 119.9, 126.5, 129.7 (2C), 131.6 (2C), 136.7, 138.1, 148.7, 149.1, 150.9, 152.1, 158.5, 160.1, 166.3, 182.8 ppm.

*7-(4-(2-(benzyloxyimino)-2-(8-methoxy-2-oxo-2H-chromen-3-yl) ethyl) piperazin-1-yl)-1-ethyl-6-fluoro-4-oxo-1, 4-dihydroquinoline-3-carboxylic acid (6k)*

White powder; M.p:(208-210) ^º^C, yield = (62%); IR (KBr, cm^-1^): 3436, 2832, 1724, 1635, 1477, 1366, 1316, 1263, 1126, 921, 758; ^1^H-NMR (500 MHz, DMSO-d6) δ =1.55 (t, 3H, CH_3_, J = 7.11 Hz), δ = 2.68-2.76 (m, 4H, piperazine), δ = 3.19-3.28 (m, 4H, piperazine), δ = 3.91 (s, 3H, O-CH_3_coumarin), δ = 3.97 (s, 2H, C-CH_2_-N), δ = 4.34 (q, 2H, CH_2_-CH_3_, J = 7.18 Hz), δ =5.30 (s, 2H, O-CH_2_-Ph), δ = 6.81 (d, 1H, H-8 quinolone , J = 6.79 Hz), δ = 7.41 (t, 1H, H-6 coumarin, J = 7.62 Hz), δ = 7.40 -7.52 (m, 5H, phenyl), δ = 7.61 -7.69 (m, 2H, H-5 and H-7 coumarin), δ = 7.94(s, 1H, H-4 coumarin), δ = 8.09 (d, 1H, H-5 quinolone, J = 12.84 Hz), δ = 8.75 (s, 1H, H-2 quinolone), δ = 15.15 (s, 1H, COOH) ppm;^ 13^C-NMR (DMSO-d6, 300 MHz): δ = 15.3, 54.8, 55.4 (2C), 57.9, 58.8 (2C), 62.3, 64.7, 80.4, 110.8, 113.55, 116.4, 119.8, 121.3, 126.7, 128.8, 129.7 (2C), 130.1 (2C), 144.2, 145.1, 146.6, 148.6, 150.3, 154.7, 158.8, 162.3, 164.8, 168.6, 172.1,174.8, 183.9 ppm. 

*7-(4-(2-(benzyloxyimino)-2-(8-methoxy-2-oxo-2H-chromen-3-yl) ethyl) piperazin-1-yl)-1-ethyl-6-fluoro-4-oxo-1, 4-dihydro-1, 8-naphthyridine-3-carboxylic acid (6l)*

White powder; M.p:(174-176) ^º^C, yield = (66%); IR (KBr, cm^-1^): 3451, 1728, 1629, 1622, 1459, 1360, 1265, 1136, 1004, 816, 744; ^1^H-NMR (500 MHz, DMSO-d6) : δ =1.52 (t, 3H, CH_3_, J = 7.11 Hz), δ = 2.61-2.74 (m, 4H, piperazine), δ = 3.63-3.81 (m, 4H, piperazine), δ = 3.90 (s, 3H, O-CH_3_coumarin), δ = 3.99 (s, 2H, C-CH_2_-N), δ =4.45 (q, 2H, CH_2_-CH_3_, J = 7.21 Hz), δ = 5.30 (s, 2H, O-CH_2_-Ph), δ = 7.41 -7.52 (m, 6H, H-6 coumarin and phenyl), 7.62 -7.73 (m, 2H, H-5 and H-7 coumarin), δ = 7.97 (s, 1H, H-4 coumarin), δ = 8.13 (d, 1H, H-5 quinolone, J = 13.24 Hz), δ =8.81 (s, 1H, H-2 quinolone), δ = 15.20 (s, 1H, COOH) ppm;^ 13^C-NMR (DMSO-d6, 300 MHz): δ = 15.9, 50.6 (2C), 52.6, 54.9, 56.4 (2C), 60.6, 63.2, 80.9, 113.8, 115.4, 118.4, 120.7, 123.3, 127.6, 129.1 (2C), 130.2 (2C), 134.7, 136.5, 139.1, 145.9, 147.1, 150.3, 153.8, 155.5, 161.9, 165.9, 170.3, 173.6, 175.9, 182.8. ppm.

*Antimicrobial and Antifungal Assay*

The antimicrobial activity was assayed by cup-plate agar diffusion method (24-25) by measuring inhibition zones in mm. *In vitro* antimicrobial activity of all synthesized compounds and standard drugs have been evaluated against two strains of bacteria which include gram-positive bacteria such as, *Bacillus subtilis* PTCC 1207 and gram-negative bacteria such as *Escherichia coli* PTCC 1047 and fungus *Candida kefyr* ATCC 38296. The antibacterial and antifungal activity was compared with standard drugs. 

The purified products were screened for their antibacterial activity by using cup-plate agar diffusion method. The nutrient agar broth prepared by the usual method, was inoculated aseptically with 0.5 mL of 24 h old subculture of, *Bacillus subtilis* PTCC 1047, and *Escherichia coli* PTCC 1047 in separate conical flasks at 40-50 ^º^C and mixed well by gently shaking. About 25 mL of the contents of the flask was poured and evenly spread in a petri dish (90 mm in diameter) and allowed to set for 2 h. The cups (10 mm in diameter) were formed by the help of borer in agar medium and filled with 0.4 mL (400μg / mL) solution of sample in DMSO.

The plates were incubated at 37 ^º^C for 24 h and the control was also maintained with 0.4 mL of DMSO in a similar manner and the zones of inhibition of the bacterial growth were measured in millimeter and recorded in (Table 2 and Figure 2).* Candida kefyr *ATCC 38296 was employed for testing antifungal activity by cup-plate agar diffusion method. The culture was maintained on Sub rouse dextrose agar slants. Sterilized Sub rouse dextrose agar medium was inoculated with 72 h old 0.5 mL suspension of fungal spores in a separate flask. About 25 mL of the inoculated medium was evenly spread on a sterilized petri dish and allowed to set for 2 h. The cups (10 mm in diameter) were punched in a petri dish and loaded with 0.4 mL (400 μg/ mL) of solution of sample in DMSO. The plates were incubated at 30 ^º^C for 48 h. After the completion of incubation period, the zones of inhibition of growth in the form of diameter in mm were measured. Along with the test solution in each petri dish one cup was filled up with solvent which acts as a control. The zones of inhibition are recorded in (Table 2 and Figure 2).

## Results and Discussion

In this study the structure of the synthesized compounds was elucidated by means of IR, ^1^H-NMR, ^13^C-NMR and Mass. All the compounds were evaluated for antibacterial and antifungal activities by cup-plate method. The antimicrobial activity of tested compounds against different strains of bacteria and fungus is shown in (Table 2 and Figure 2). The newly synthesized compounds 6a–l were evaluated for their *in-vitro* antibacterial activity against *Bacillus subtilis* PTCC 1207, *Escherichia coli* PTCC 1047 and *Candida kefyr* ATCC 38296 using conventional by cup-plate agar diffusion method (24). The zone of growth inhibition values was determined by comparison to standard drugs. The zone of growth inhibition obtained for compounds 6a–l are presented in (Table 2 and Figure 2).

 The zone of growth inhibition values of the test derivatives indicated that most compounds exhibit good activity against gram-positive and gram-negative bacteria. Antibacterial screening of compounds 6a–l against gram-positive and gram-negative bacteria reveals that compounds 6g, 6h and 6i exhibit the most potent *in-vitro* antibacterial activity. All compounds show improvement of activity against gram-positive bacteria in comparison to standard drugs. Generally, in both gram-positive and gram-negative bacteria, better results are obtained with cyclopropyl and ethyl at the N-1position of the quinolone ring and 2-NOCH_3_ on the ethyl spacer of coumarin and piperazine rings.

## Conclusion

some of the new N-[2-(coumarin-3-yl)ethyl]piperazinyl quinolones 6 containing a carbonyl related functional groups (ketone, oxime, *O*-methyloxime, and *O*-benzyloxime) on the ethyl spacer showed considerable antibacterial activity and modification of the position 8 and N-1 substituent on the quinolone ring, and ethyl spacer functionality produced relatively major changes in terms of activity. In general, the results of antibacterial evaluation of the test compounds in comparison with the reference drugs indicated that compounds6g, 6 h and 6i showed comparable or more potent antibacterial activity with respect to the reference drugs against all tested species. The antifungal data reveals that all compounds have shown weak antifungal activity as compared to 

Nistatin. 
